# Supplementary material for: The telomere length landscape of prostate cancer
Source: Nat Commun. 2021 Nov 25;12:6893. doi: 10.1038/s41467-021-27223-6 (PMC8617305; doi:10.1038/s41467-021-27223-6)
Supplement: Supplementary file 1 — Supplementary Information [file 41467_2021_27223_MOESM1_ESM.pdf]

Supplementary Information

The Telomere Length Landscape of Prostate Cancer

Julie Livingstone[1,2,3,4], Yu-Jia Shiah[5], Takafumi N. Yamaguchi[1,2,3,4], Lawrence E. Heisler[5], Vincent Huang[5], Robert Lesurf[5], Tsumugi Gebo[1,2,3,4], Benjamin Carlin[1,2,3,4], Stefan Eng[1,2,3,4], Erik Drysdale[5], Jeffrey Green[5], Theodorus van der Kwast[6,7], Robert G. Bristow[6,8,9], Michael Fraser[6], Paul C. Boutros[*,1,2,3,4,8,10]

[1] Department of Human Genetics, University of California, Los Angeles, CA 90095, USA
[2] Department of Urology, University of California, Los Angeles, CA 90024, USA
[3] Jonsson Comprehensive Cancer Centre, University of California, Los Angeles, CA 90024, USA
[4] Institute for Precision Health, University of California, Los Angeles, CA 90024, USA
[5] Ontario Institute for Cancer Research, Toronto, ON M5G 0A3, Canada
[6] Princess Margaret Cancer Centre, University Health Network, Toronto, ON M5G 2M9, Canada
[7] Department of Pathology, Laboratory Medicine Program, University Health Network, Toronto, ON M5G 2C4, Canada
[8] Department of Medical Biophysics, University of Toronto, Toronto, ON M5G 1L7, Canada
[9] Manchester Cancer Research Centre, Manchester, United Kingdom
[10] Department of Pharmacology and Toxicology, University of Toronto, Toronto, ON M5S 1A8, Canada
*Corresponding Author

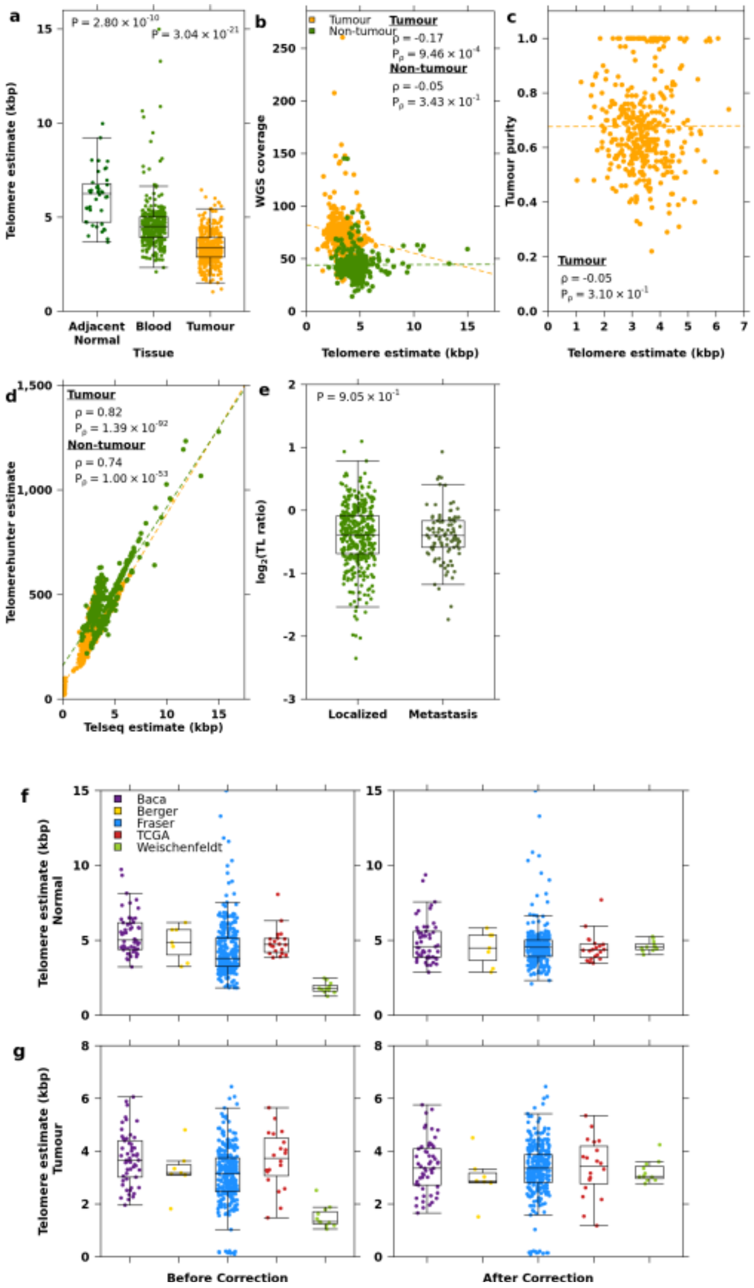

Supplementary Figure 1 | Telomere length is independent of technical variables. **a,** Comparison of telomere length (TL) in adjacent, histologically normal prostate tissue (n = 40), blood (n = 341), and tumour tissue (n = 381). *P* values are from a two-sided Mann Whitney U-test comparing adjacent normal TL to blood TL and tumour TL. **b,** Two-sided Spearman's correlation of telomere length (n = 381) estimated by TelSeq and WGS coverage **c,** tumour purity and **d,** TelomereHunter estimates. **e,** Comparison of TL ratio in localized (n = 381) and metastatic (n = 101) prostate cancer samples. *P* value is from a two-sided Mann-Whitney U test. **f,** Non-tumour TL and **g,** tumour TL were batch corrected using a linear model (see Methods). Pre-corrected values and corrected values are shown. Box plots depict the upper and lower quartiles, with the median shown as a solid line; whiskers indicate 1.5 times the interquartile range (IQR).

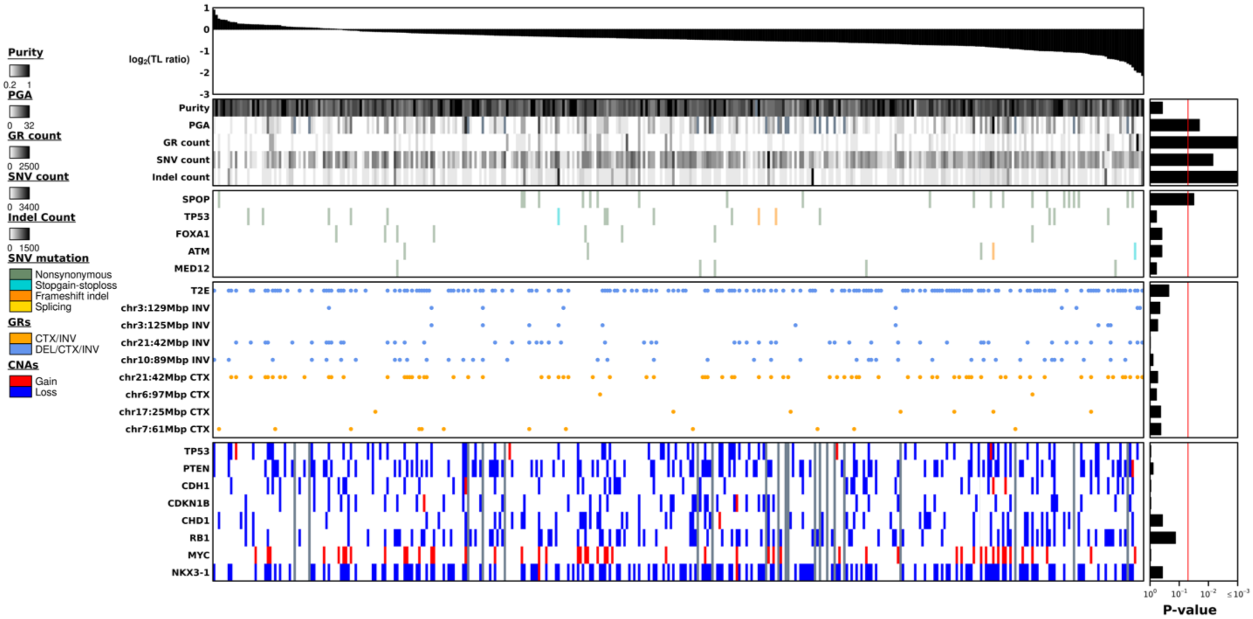

Supplementary Figure 2 | Genomic associations with telomere length (TL) ratio. TL ratio (tumour TL / non-tumour TL) is ranked in descending order. The association of TL ratio and measures of mutational burden, TMPRSS2:ERG (T2E) fusion status, as well as, known prostate cancer genes with recurrent copy number aberrations (CNAs), coding single-nucleotide variants (SNVs), and genomic rearrangements (GRs) are shown. Bar plots indicate the statistical significance of each association (see Methods**)**.

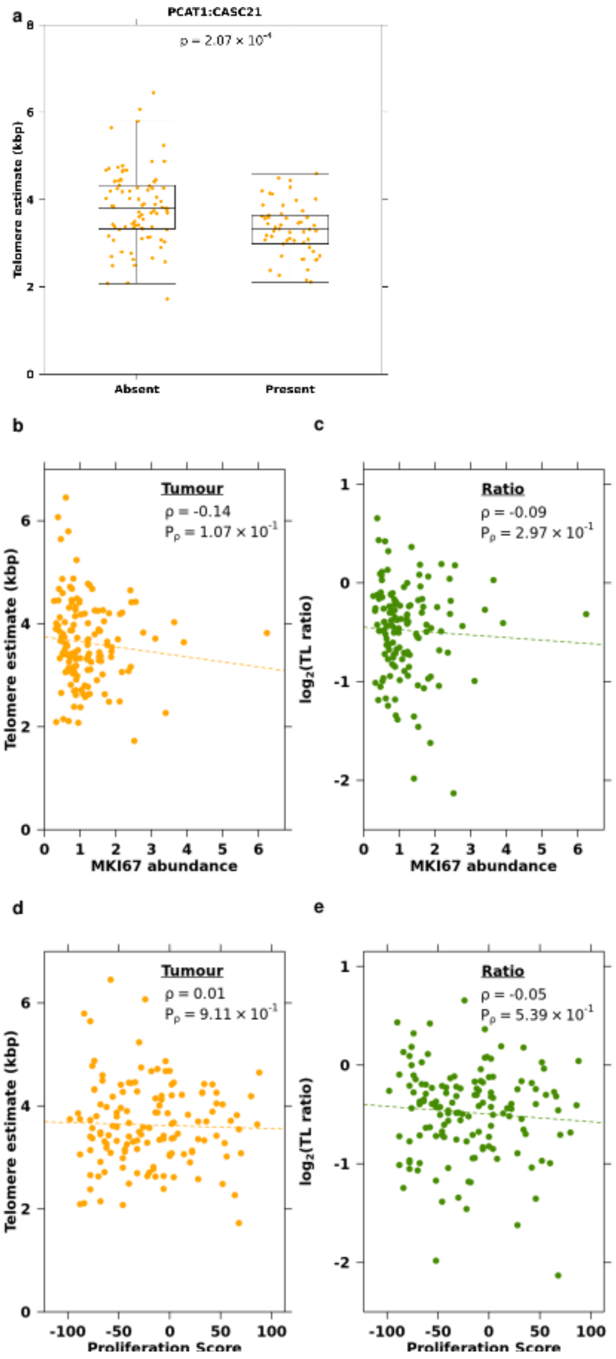

Supplementary Figure 3 | Fusions are association with tumour TL and TL ratio.
a, Difference in tumour TL between samples with a PCAT1:CASC21 gene fusion and those without. Box plots depict the upper and lower quartiles, with the median shown as a solid line; whiskers indicate 1.5 times the interquartile range (IQR). **b-c,** Correlation of MKI57 RNA abundance with **b,** tumour TL and **c,** TL ratio. **d-e,** Correlation of proliferation scores with **d,** tumour TL and **e,** TL ratio. Two-sided Spearman's ρ and *P* values are displayed.

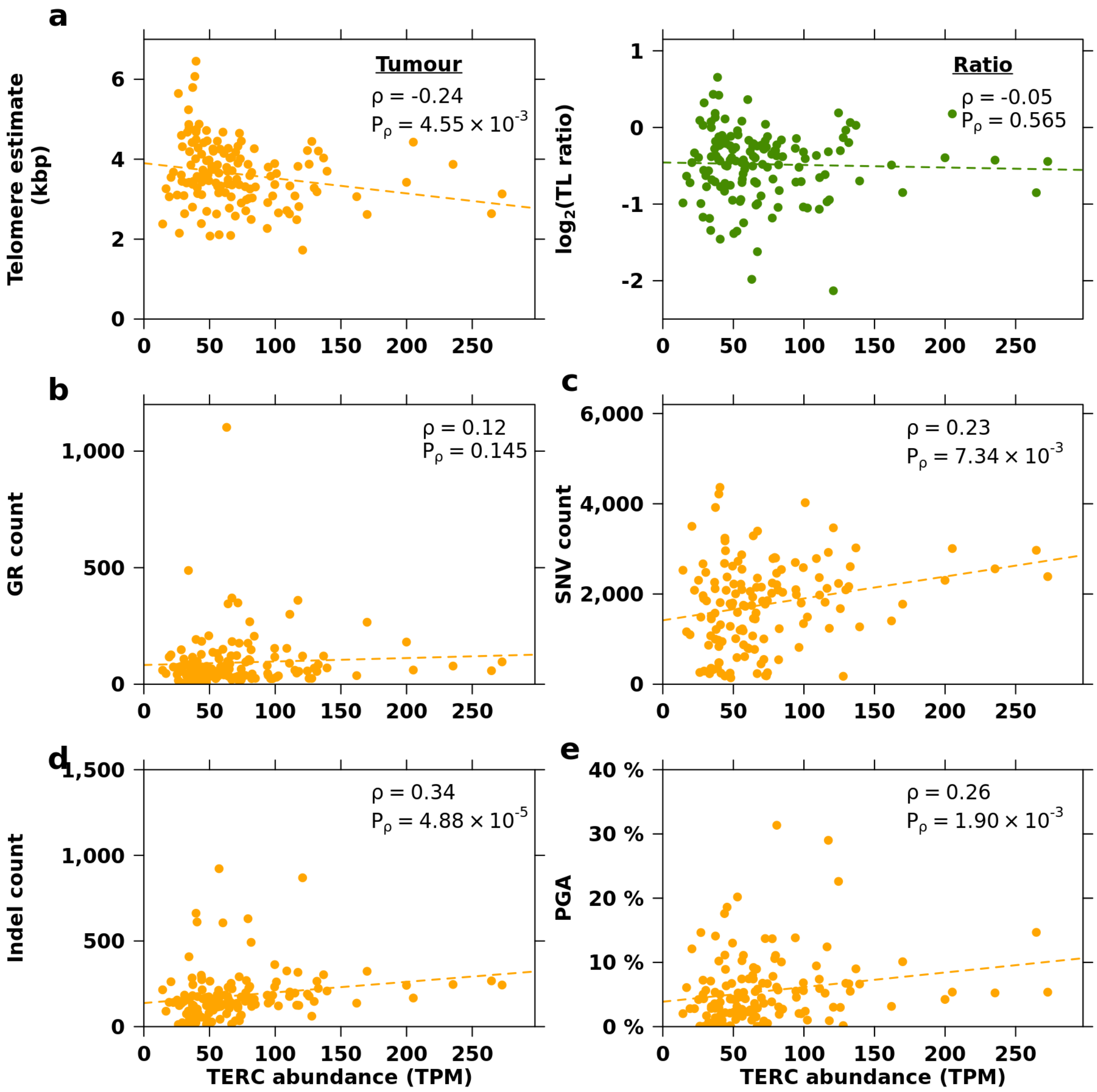

Supplementary Figure 4 | Genomic correlates of TERC abundance.
a, Correlation of *TERC* abundance with tumour TL and TL ratio. **b-e,** Correlation of *TERC* abundance and the **b,** number of genomic rearrangements (GRs), **c,** number of single-nucleotide variants (SNVs), **d,** number of indels and **e,** percent genome altered (PGA). Two-sided Spearman's ρ and *P* values are displayed (n = 139).

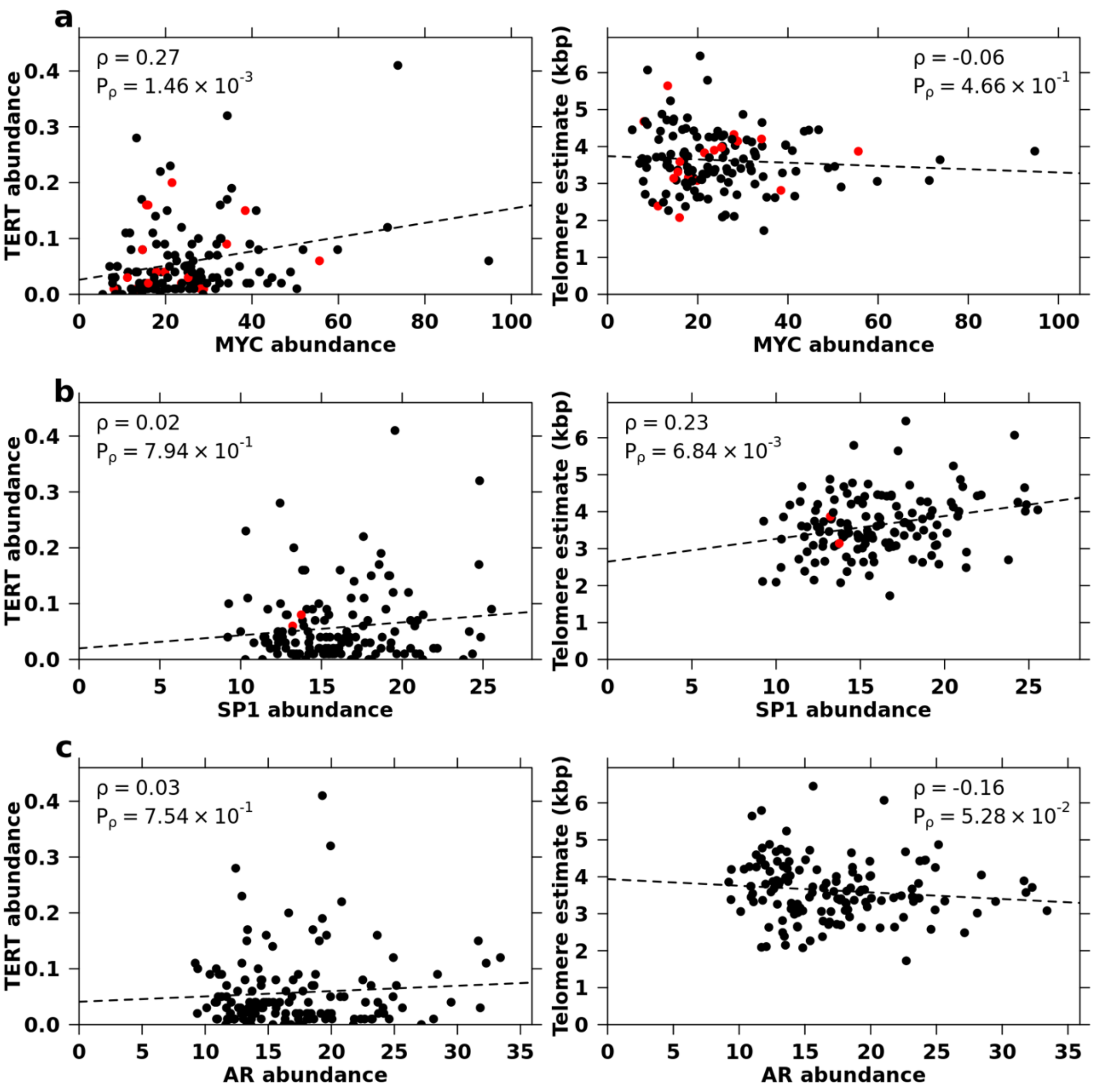

Supplementary Figure 5 | Transcription factors of *TERT* are correlated with *TERT* abundance and telomere length (TL).

a-c, Correlation of *TERT* transcription factors **a,** *MYC,* **b,** *SP1* and **c,** *AR* with *TERT* abundance and tumour TL. Two-sided Spearman's ρ and *P* values are displayed. Red dots indicate an amplification in that sample for the displayed transcription factor.

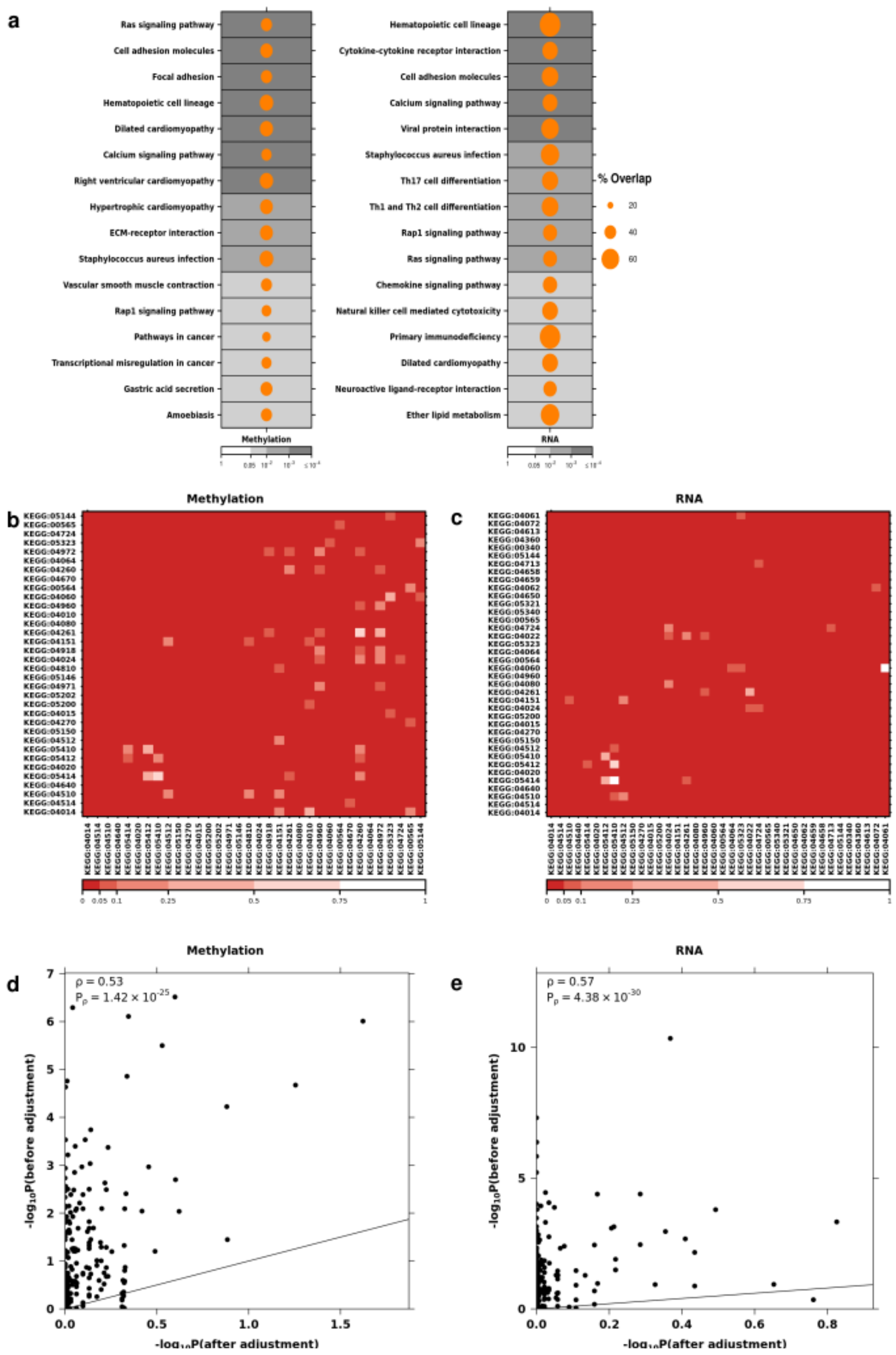

Supplementary Figure 6 | Pathways enriched in genes with methylation or transcriptomic profiles that are correlated with tumour TL. a, Dotmap representing enriched pathways. Size of dot indicates the percentage of overlap between correlated genes and genes in the pathway. Background colour indicates unadjusted *P* values from gprofiler2. **b-c**, Heatmaps of crosstalk matrices where white indicates loss of significance after removal of intersecting genes. **d-e,** Comparison of *P* values from an one-sided Fisher's Exact test before and after crosstalk adjustment.

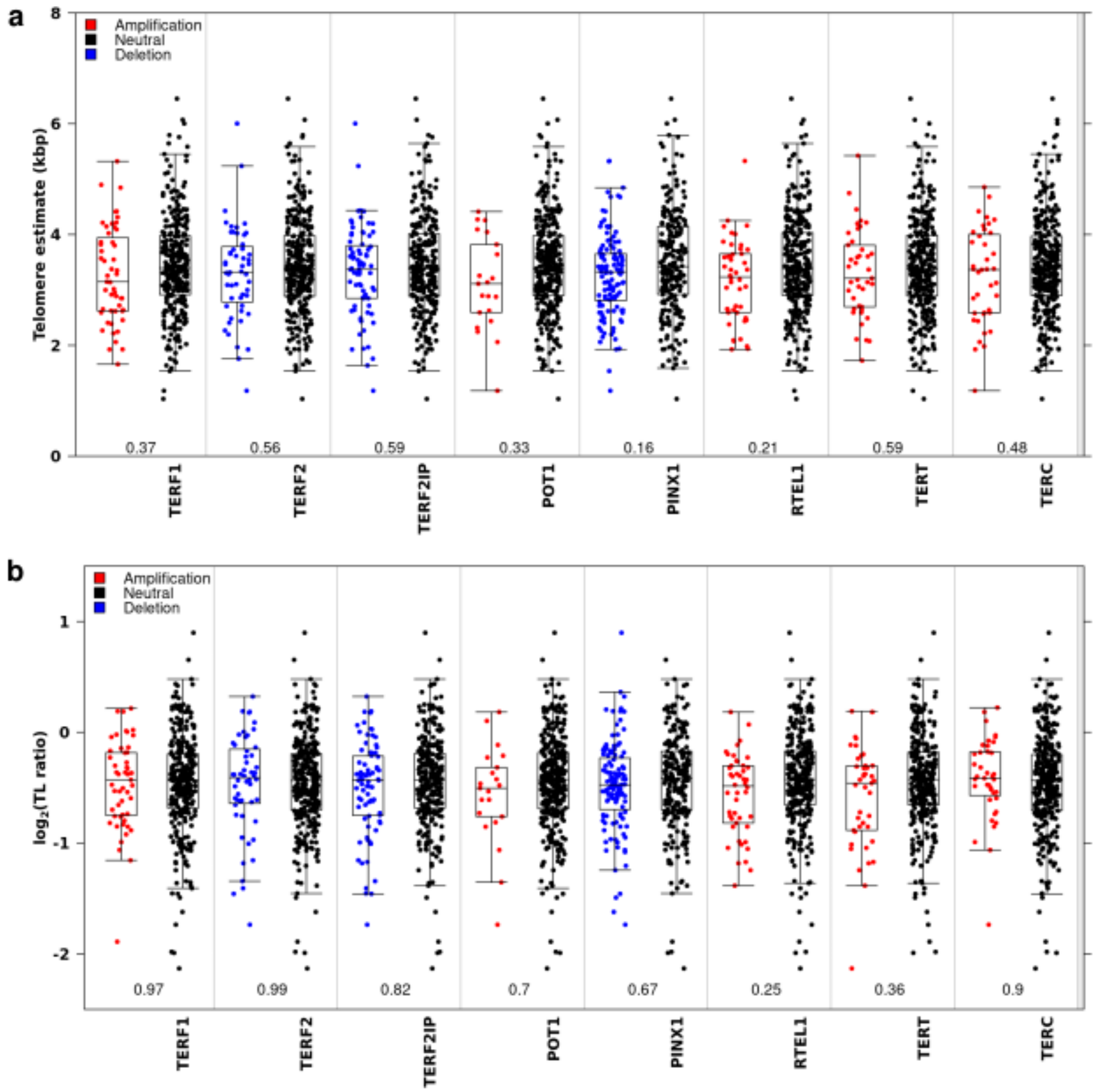

Supplementary Figure 7 | Telomere length (TL) does not differ by copy number status in genes that make up the telomere complex.

a-b, Difference in **a,** tumour TL and **b,** TL ratio between samples (n = 381) with a copy number aberration and those without in telomere complex genes. *Q* values are from a two-sided Mann-Whitney U test. Box plots depict the upper and lower quartiles, with the median shown as a solid line; whiskers indicate 1.5 times the interquartile range (IQR).

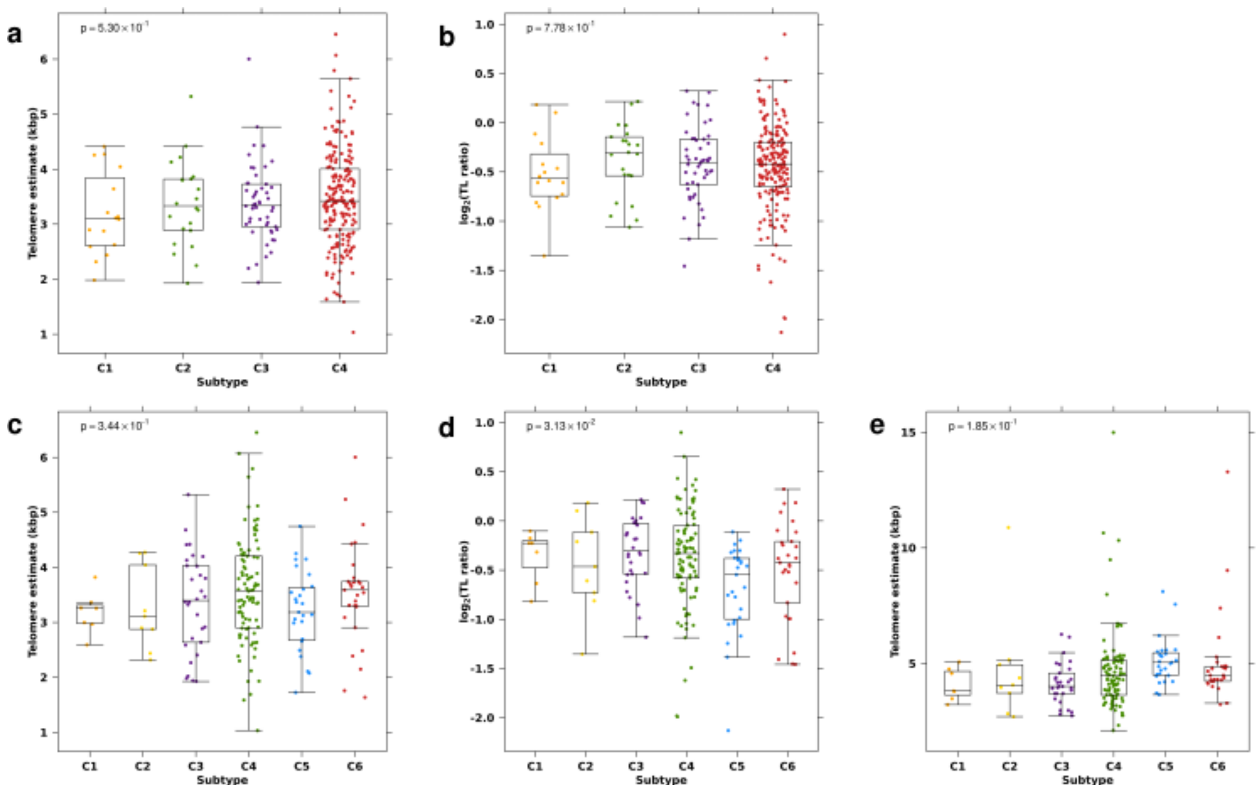

Supplementary Figure 8 | Association of telomere length (TL) with CNA subtypes. a-b, Association of **a,** tumour TL and **b,** TL ratio with four previously identified copy number aberration subtypes[1]. *P* value is from a two-way ANOVA (n = 284). **c-e,** Association of **c,** tumour TL and **d,** TL ratio and **e,** non-tumour TL with seven previously identified CNA subtypes[2]. *P* value is from a two-way ANOVA (n = 196). Box plots depict the upper and lower quartiles, with the median shown as a solid line; whiskers indicate 1.5 times the interquartile range (IQR).

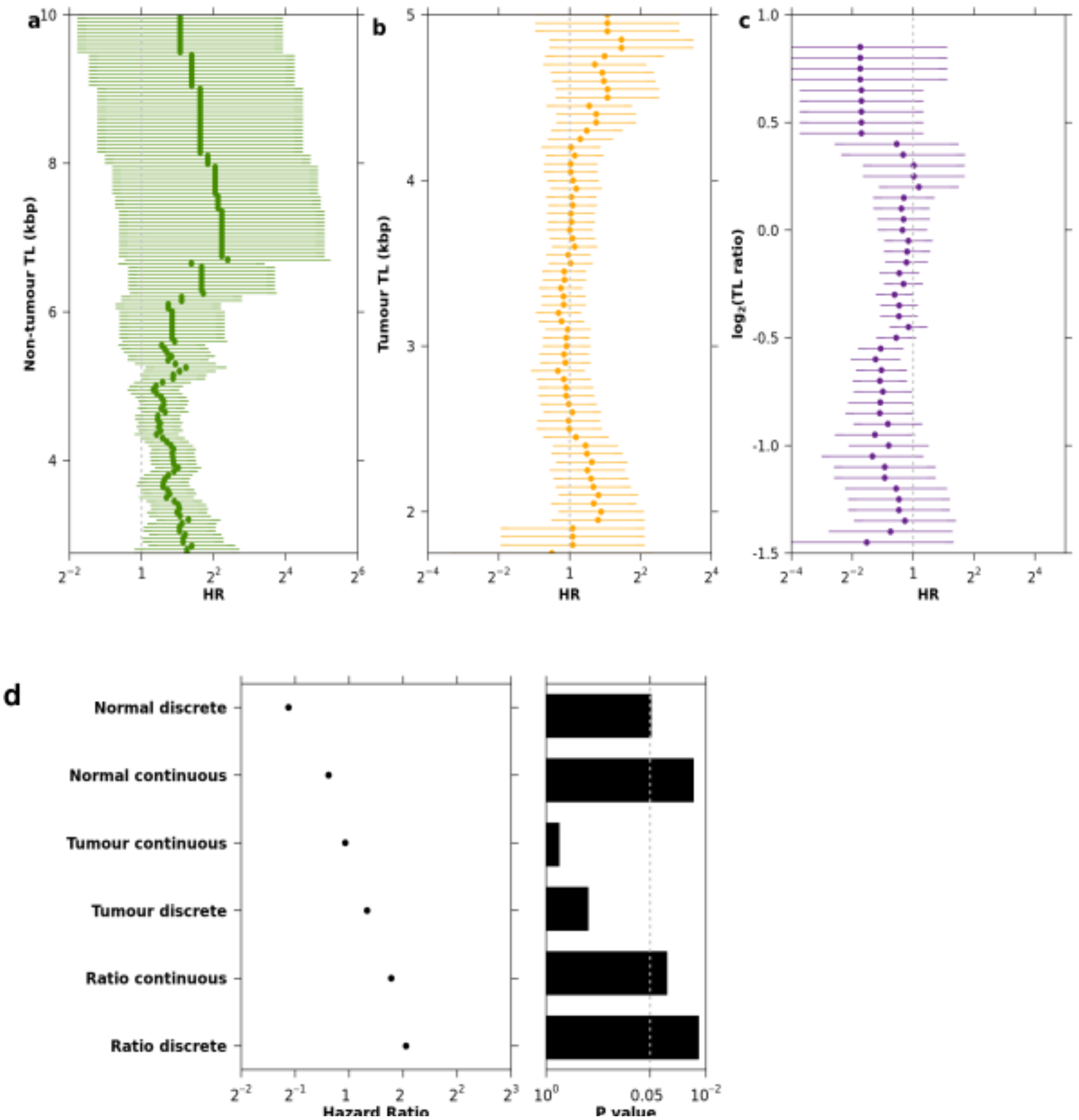

Supplementary Figure 9 | Telomere length (TL) is associated with biochemical relapse.

a-c, Association of **a,** non-tumour TL, **b,** tumour TL and **c,** TL ratio with biochemical relapse using a Cox proportional hazards model (n = 290) at different TL cutoffs, incremented by 50 bp. Error bars represent the 95% confidence interval (HR = hazard ratio). **d,** Comparison of the best dichotomized Cox proportional hazards models and models fit with TL as a continuous value.

Supplementary References

1. Lalonde, E. *et al.* Tumour genomic and microenvironmental heterogeneity for integrated prediction of 5-year biochemical recurrence of prostate cancer: a retrospective cohort study. *Lancet Oncol.* **15**, 1521–1532 (2014).
2. Fraser, M. *et al.* Genomic hallmarks of localized, non-indolent prostate cancer. *Nature* **541**, 359–364 (2017).
