# Supplementary material for: The telomere length landscape of prostate cancer
Source: Nat Commun. 2021 Nov 25;12:6893. doi: 10.1038/s41467-021-27223-6 (PMC8617305; doi:10.1038/s41467-021-27223-6)
Supplement: Supplementary file 3 — Description of Additional Supplementary Files [file 41467_2021_27223_MOESM3_ESM.pdf]

File Name: SupplementaryData1.xlsx

Description: Clinical data for 381 samples used in analysis after applying quality control metrics.

File Name: SupplementaryData2.xlsx

Description: Statistical summary of 47 recurrent fusions pairs tested for association with TL using a two-sided Mann-Whitney U test.

File Name: SupplementaryData3.xlsx

Description: Results from two-sided Spearman's correlation between tumour TL and methylation beta values, RNA abundance and protein abundance. *Q* values are FDR adjusted *P* values. NAs indicate missing values where tests could not be performed.

File Name: SupplementaryData4.xlsx

Description: Results from two-sided Spearman's correlation between TL ratio (tumour TL/ non-tumour TL) and methylation beta values, RNA abundance and protein abundance. *Q* values are FDR adjusted *P* values. NAs indicate missing values where tests could not be performed.

File Name: SupplementaryData5.xlsx

Description: Associations between CNAs and Tumour TL, ordered by *Q* values. Each row represents collapsed segments containing multiple genes. Contiguous gene segments with aberrations in less that 5% of patients were removed.

File Name: SupplementaryData6.xlsx

Description: Statistically significant associations between CNAs and TL ratio (tumour TL/non-tumour TL), ordered by *Q* values. Each row represents collapsed segments containing multiple genes. Contiguous gene segments with aberrations in less that 5% of patients were removed.
