# Supplementary material for: The telomere length landscape of prostate cancer
Source: Nat Commun. 2021 Nov 25;12:6893. doi: 10.1038/s41467-021-27223-6 (PMC8617305; doi:10.1038/s41467-021-27223-6)
Supplement: Supplementary file 10 — Reporting Summary [file 41467_2021_27223_MOESM10_ESM.pdf]

nature portfolio

Corresponding author(s): Paul C. Boutros

Last updated by author(s): November 3rd 2021

Reporting Summary

Nature Portfolio wishes to improve the reproducibility of the work that we publish. This form provides structure for consistency and transparency in reporting. For further information on Nature Portfolio policies, see our Editorial Policies and the Editorial Policy Checklist.

Statistics

For all statistical analyses, confirm that the following items are present in the figure legend, table legend, main text, or Methods section.

n/a	Confirmed	
☐	☒	The exact sample size (*n*) for each experimental group/condition, given as a discrete number and unit of measurement
☐	☒	A statement on whether measurements were taken from distinct samples or whether the same sample was measured repeatedly
☐	☒	The statistical test(s) used AND whether they are one- or two-sided<br>*Only common tests should be described solely by name; describe more complex techniques in the Methods section.*
☐	☒	A description of all covariates tested
☐	☒	A description of any assumptions or corrections, such as tests of normality and adjustment for multiple comparisons
☐	☒	A full description of the statistical parameters including central tendency (e.g. means) or other basic estimates (e.g. regression coefficient) AND variation (e.g. standard deviation) or associated estimates of uncertainty (e.g. confidence intervals)
☐	☒	For null hypothesis testing, the test statistic (e.g. *F*, *t*, *r*) with confidence intervals, effect sizes, degrees of freedom and *P* value noted<br>*Give P values as exact values whenever suitable.*
☒	☐	For Bayesian analysis, information on the choice of priors and Markov chain Monte Carlo settings
☒	☐	For hierarchical and complex designs, identification of the appropriate level for tests and full reporting of outcomes
☐	☒	Estimates of effect sizes (e.g. Cohen's *d*, Pearson's *r*), indicating how they were calculated

Our web collection on statistics for biologists contains articles on many of the points above.

Software and code

Policy information about availability of computer code

Data collection	No software was used
Data analysis	qpure (v1.1), BWA-mem (> v0.7.12), Picard (v1.92), GATK (> v3.4.0), SAMtools (v0.1.9), TelSeq (v0.0.1), BAMQL (v1.6), TelomereHunter (v1.0.4), STAR v2.5.3a), RSEM (v1.2.29), SomaticSniper (v1.0.5), Delly (v0.7.8), BEDtools (v2.17.0), OncoScan Console (v1.1), gprofiler2(0.2.1), ShatterProof (v0.14), R (v4.1.1), BPG (BoutrosLab.Plotting.General; v5.6.1), survival (v3.2-7), wateRmelon (v1.15.1), DMAcate(v1.4.2), IlluminaHumanMethylation450kanno.ilmn12.hg19 package (v0.6.0)

For manuscripts utilizing custom algorithms or software that are central to the research but not yet described in published literature, software must be made available to editors and reviewers. We strongly encourage code deposition in a community repository (e.g. GitHub). See the Nature Portfolio guidelines for submitting code & software for further information.

Data

Policy information about availability of data

All manuscripts must include a data availability statement. This statement should provide the following information, where applicable:

- Accession codes, unique identifiers, or web links for publicly available datasets
- A description of any restrictions on data availability
- For clinical datasets or third party data, please ensure that the statement adheres to our policy

The publicly available WGS data used in this study are available in the EGA and dbGaP databases under the following accession codes; Baca: phs000447.v1.p1 [https://www.ncbi.nlm.nih.gov/projects/gap/cgi-bin/study.cgi?study_id=phs000447.v1.p1], Berger: phs000330.v1.p1 [https://www.ncbi.nlm.nih.gov/projects/gap/cgibin/study.cgi?study_id=phs000330.v1.p1], ICGC PRAD-CA: EGAS00001000900 [https://ega-archive.org/studies/EGAS00001000900], TCGA PRAD: phs000178.v11.p8 [https://www.ncbi.nlm.nih.gov/projects/gap/cgi-bin/study.cgi?study_id=phs000178.v11.p8], EO-PCA: EGAS00001000400 [https://ega-

archive.org/studies/EGAS00001000400], WCDT-MCRPC: phs001648.v2.p1 [https://www.ncbi.nlm.nih.gov/projects/gap/cgi-bin/study.cgi?study_id=phs001648.v2.p1. These data are available under controlled access after authorization by a Data Access Committee. Access can be requested via EGA or dbGaP. Publicly available processed variant calls (CNAs, GRs, SNVs, indels) are available through the ICGC Data Portal under the project PRAD-CA (https://dcc.icgc.org/projects/PRAD-CA). The publicly available OncoScan SNP array data and RNA-Sequencing data used in this study can be found on EGA under the accession EGAS00001000900 [https://ega-archive.org/studies/EGAS00001000900]. The publicly available mRNA data used in this study is available in the Gene Expression Omnibus (GEO) database under the accession GSE84043 [https://www.ncbi.nlm.nih.gov/geo/query/acc.cgi?acc=GSE84043]. The publicly available methylation data used in this study is available in the GEO database under the accession GSE107298 [https://www.ncbi.nlm.nih.gov/geo/query/acc.cgi?acc=GSE107298]. The publicly available processed proteomics data are available in supplementary material online [https://doi.org/10.1016/j.ccell.2019.02.005]. The publicly available processed RNA-Seq data and gene fusion data are available in supplementary material online [https://doi.org/10.1016/j.cell.2019.01.025]. The data generated in this study, including tumour and non-tumour telomere lengths and association statistics, are available within the article or Supplementary Information.

Field-specific reporting

Please select the one below that is the best fit for your research. If you are not sure, read the appropriate sections before making your selection.

- [x] Life sciences
- [ ] Behavioural & social sciences
- [ ] Ecological, evolutionary & environmental sciences

For a reference copy of the document with all sections, see nature.com/documents/nr-reporting-summary-flat.pdf

Life sciences study design

All studies must disclose on these points even when the disclosure is negative.

Sample size	392 published WGS from intermediate risk prostate cancer tumour blood or adjacent normal pairs. RNA abundance was available for 139 samples, methylation data was available for 241 samples and proteomics data was available for 70 samples. 101 published WGS from metastatic prostate cancer tumour-blood pairs. This is a collection of publicly available datasets with WGS. Other data type are from the ICGC PRAD-CA, which consists of different data types from the same patient (i.e. methylation, RNA-seq, proteomics)
Data exclusions	Reads from lanes that contained too few reads to calculate an estimate (marked as UNKNOWN), and outlier lanes as identified by grub's test, were removed from input BAMs using BAMQL v1.6. These outliers were removed due to technical failures. After outliers were removed, TelSeq was run again ignoring read groups with the -u parameter. Samples with telomere estimates less than 0.25 were removed from further analysis. (n = 11). These samples were removed because their values were four standard deviations lower than the mean and thought to be due to technical issues and not biologically sound.
Replication	In order to replicate our findings, we ran analyses using telomere estimates from two different algorithms. A substantially powered intermediate risk prostate cancer validation dataset with WGS, methylation, transcriptomics and proteomics data doesn't exist.
Randomization	Samples were allocated to groups based telomere length or mutation status depending on the test. To account for differences in TL due to sequencing center, a linear model was fit with TL as the response variable and sequencing center as the predictor variable
Blinding	All participants were recruited into the study based only on clinical features. Therefore, telomere length nor somatic mutational profile was known upon inclusion. Blinding was not relevant in this study because we were not comparing treatment groups.

Reporting for specific materials, systems and methods

We require information from authors about some types of materials, experimental systems and methods used in many studies. Here, indicate whether each material, system or method listed is relevant to your study. If you are not sure if a list item applies to your research, read the appropriate section before selecting a response.

Materials & experimental systems

n/a	Involved in the study	
x		Antibodies
x		Eukaryotic cell lines
x		Palaeontology and archaeology
x		Animals and other organisms
	x	Human research participants
x		Clinical data
x		Dual use research of concern

Methods

n/a	Involved in the study	
x		ChIP-seq
x		Flow cytometry
x		MRI-based neuroimaging

Human research participants

Policy information about studies involving human research participants

Population characteristics	All relevant population characteristics have been provided in Supplementary Data 1
Recruitment	All patients had a prostate and N0M0 prostate cancer as an entry criteria for the study.
Ethics oversight	Informed consent, consistent with the guidelines of the local Research Ethics Board (REB) and International Cancer Genome Consortium (ICGC), was obtained at the time of clinical follow-up. Previously collected tumour tissues were used, following University Health Network REB-approved study protocols (UHN 06-0822-CE, UHN 11-0024-CE, CHUQ 2012-913:H12-03-192) and IRB #21-009599. Participants were not compensated for their involvement.

Note that full information on the approval of the study protocol must also be provided in the manuscript.
